# Maternal Uniparental Isodisomy of Chromosome 2 Leading to Homozygous Variants in *SPR* and *ZNF142* : A Case Report and Review of the UPD2 Literature

**DOI:** 10.1055/s-0044-1785442

**Published:** 2024-03-26

**Authors:** Janhawi Kelkar, Miriam DiMaio, Deqiong Ma, Hui Zhang

**Affiliations:** 1 Division of Medical Genetics and Genomics, Icahn School of Medicine at Mount Sinai, New York, New York, United States; 2 Department of Genetics, Yale School of Medicine, New Haven, Connecticut, United States

**Keywords:** chromosome 2 uniparental disomy, sepiapterin reductase deficiency, genomic imprinting

## Abstract

We report a 4-year-old girl with neurodevelopmental abnormalities who has maternal uniparental isodisomy of chromosome 2 leading to homozygosity for a likely pathogenic variant in *SPR* , and a variant of uncertain significance in *ZNF142* . Biallelic pathogenic variants in *SPR* lead to sepiapterin reductase deficiency (SRD), a dopa-responsive dystonia. Pathogenic variants in *ZNF142* are associated with an autosomal recessive neurodevelopmental disorder characterized by impaired speech and hyperkinetic movements, which has significant clinical overlap with SRD. Our patient showed dramatic improvement in motor skills after treatment with levodopa. We also reviewed 67 published reports of uniparental disomy of chromosome 2 (UPD2) associated with various clinical outcomes. These include autosomal recessive disorders associated with loci on chromosome 2, infants with UPD2 whose gestations were associated with confined placental mosaicism for trisomy 2 leading to intrauterine growth restriction with good postnatal catchup growth, and normal phenotypes in children and adults with an incidental finding of either maternal or paternal UPD2. These latter reports provide support for the conclusion that genes located on chromosome 2 are not subject to imprinting. We also explore the mechanisms giving rise to UPD2.

## Introduction

Uniparental disomy (UPD) is the inheritance of two homologous chromosomes or two homologous segments of a chromosome from one parent. This hypothetical concept was first proposed by Engel in 1980, who recognized that the high frequency of aneuploidy in gametes made it possible for two aneuploid gametes to produce a euploid zygote. 1

UPD is often associated with a meiotic nondisjunction event leading to an aneuploid gamete followed by a mitotic nondisjunction event in the conceptus. The most common cause of UPD is known as trisomy rescue, where a mitotic nondisjunction event in a trisomic conceptus leads to restoration of euploidy in most or all cell lineages. 2 A less common cause of UPD is gamete compensation where a monosomic embryo is rescued by duplication of the monosomic chromosome. Other rare mechanisms have also been reported, including gamete complementation where a disomic and nullisomic gamete unite, as initially proposed by Engel. UPD can involve a whole chromosome or a chromosomal segment.

Failure of homologous chromosomes to separate in meiosis I followed by a postzygotic trisomy rescue event can lead to the inheritance of two nonidentical chromosomes from the same parent, known as heterodisomy. Failure of sister chromatids to separate in meiosis II followed by a trisomy rescue event can lead to the inheritance of two identical chromosomes from the same parent is known as isodisomy.

The incidence of UPD for any chromosome was initially reported to be 1 in 3,500. 3 A recent study of 32,067 whole exome sequencing trios found an overall prevalence of UPD of 1 in 500. 4 In the setting of a parent who carries a balanced Robertsonian translocation, the risk of UPD in a child who inherits the balanced translocation is estimated at 1 in 150. 5

UPD may have adverse effects on development for several reasons. These include the inheritance of two pathogenic variants in a gene from the same parent resulting in an autosomal recessive disorder, or an imprinting disorder due to lack of expression of a functional gene. If UPD is associated with a trisomy or monosomy rescue event during early embryogenesis, there also could be adverse effects on development related to an aneuploid cell line in the fetus which may be cryptic, and/or aneuploidy in the placenta.

We report the first case of maternal uniparental isodisomy of chromosome 2 leading to homozygosity for variants in the genes *SPR* and *ZNF142* . *SPR* is located on chromosome 2p13.2 which encodes sepiapterin reductase, an enzyme involved in the biosynthesis of tetrahydrobiopterin (BH _4_ ), a cofactor in the synthesis of monoamine neurotransmitters. Pathogenic variants in this gene lead to sepiapterin reductase deficiency (SRD), a dopa-responsive dystonia with autosomal recessive inheritance. *ZNF142* is located on chromosome 2q35, encodes a zinc finger transcription factor with increased expression in the cerebellum. Pathogenic variants in *ZNF142* have recently been associated with a recessive neurodevelopmental disorder characterized by impaired speech and hyperkinetic movements (NEDISHM).

We also reviewed 66 published reports of uniparental disomy of chromosome 2 (UPD2) which has been observed in association with various autosomal recessive disorders with loci on chromosome 2, confined placental mosaicism for trisomy 2 leading to intrauterine growth restriction (IUGR) with good postnatal catch-up growth, and normal phenotypes in children and adults with an incidental finding of either maternal or paternal UPD2. These latter reports provide preliminary support for the conclusion that genes located on chromosome 2 are not subjected to imprinting. Lastly, we explore the mechanisms giving rise to UPD2.

## Case Presentation

A 4-year 2-month-old girl was referred to genetics clinic for evaluation of developmental delay and abnormal eye movement. She was accompanied by her parents.

The patient was born at 41 weeks' gestation to a 24-year-old gravida 1 para 0 mother and a 28-year-old father after an uncomplicated pregnancy and vaginal delivery. Family history of the patient was noncontributory. Consanguinity was denied.

Her birth weight was 3.3 kg (50th percentile), and her length was reportedly average. Information about Apgar scores was not available. Her immediate postnatal course was unremarkable.

At about the age of 3 months, frequent upward deviation of her eyes was noted. An electroencephalogram (EEG) was performed, which did not capture seizures. At the age of 5 months, the patient was hospitalized for seizure-like activity, and she was treated with anticonvulsants. Workup included an EEG which was reportedly normal, and a brain MRI at the age of 6 months which did not identify specific abnormalities. At 7.5 months, her parents took her home from the hospital and stopped all medication due to lack of improvement in symptoms.

Shortly after, another EEG was performed at a third hospital, which did not capture seizures. She was evaluated by an ophthalmologist who diagnosed her with vertical periodic nystagmus. She was also evaluated by a clinical geneticist who ordered biochemical testing. Urine organic acids and plasma amino acids were reportedly normal.

The patient had global developmental delays. She sat unassisted at 1.5 years of age, and she took independent steps at 4 years of age. At the time of genetic evaluation, her speech was limited to a few single syllables, and she was able to use made-up hand signs to communicate with her family. Her receptive language was better than expressive language. The family moved to the United States at 3 years 9 months to seek medical care. In the United States, she started to receive occupational therapy, physical therapy, and speech therapy, which helped her make developmental progress. Repeated brain MRI was also ordered in the United States, which was reportedly normal.

On physical examination, she held a chin-up position and her eyes rolled back intermittently. Head circumference was 48 cm (15th percentile), height was 100.9 cm (40th percentile), and weight was 15.5 kg (37th percentile). No significant dysmorphic features were noted. Her musculoskeletal exam revealed bilateral pes planus and joint laxity. Her neurologic exam showed brisk reflexes, generalized truncal hypotonia, and unsteady gait. Nystagmus was not noted.

**Fig. 1:**
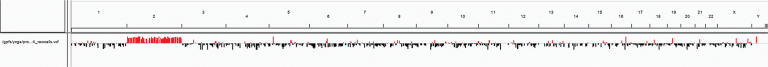
This graph shows areas with loss of homozygosity across the genome of the proband. Heterozygous variants are in black and homozygous variants are in red. Only homozygous variants in red are observed across the entire chromosome 2.

**Fig. 2:**
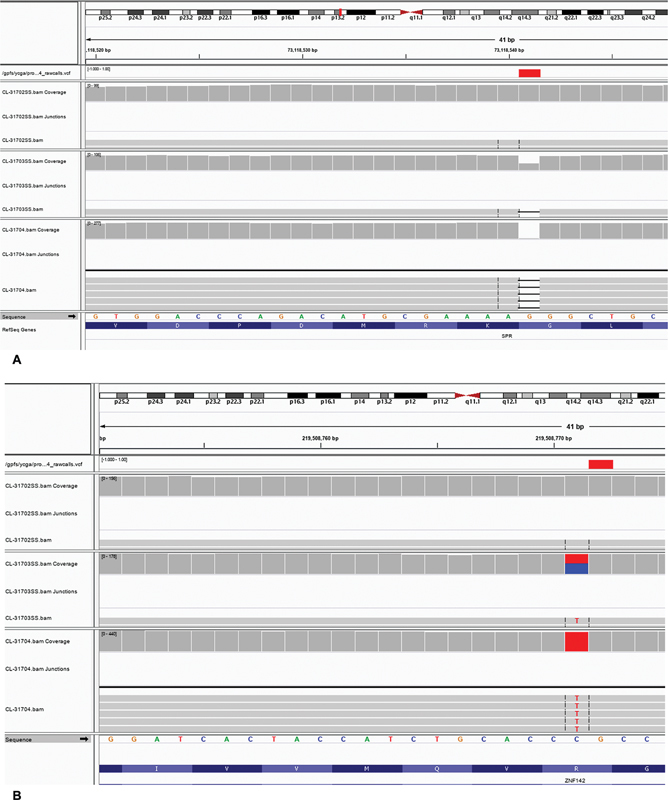
( **A** ) SPR variants from the proband, and the parents in the Integrative Genomics Viewer (IGV), demonstrating the proband is homozygous and the mother is heterozygous for the variant and the father does not carry the variant. Variant: SPR:NM_003124:exon3:c.661delG:p.p.Leu222CysfsTer4 (chr2:73118540) [hg19]. The read count of the variant in proband is 172 (homozygous) and is 33 out of 66 in the mother (heterozygous). The father does not carry the variant. ( **B** ) ZNF142 variants from the proband, and the parents in the IGV, demonstrating the proband is homozygous and the mother is heterozygous for the variant and the father does not carry the variant. Variant: ZNF142:NM_001105537:exon8:c.G2468A:p.Arg823Gln (chr2:219508771) [hg19]. The read count of the variant in proband 38 39 40 is 268 (homozygous) and is 61 out of 134 in the mother (heterozygous). The father does not carry the variant.

Whole exome sequencing analysis revealed excessive homozygous rare variants on chromosome 2 ( Fig. 1 ). A detailed evaluation of the variants on chromosome 2 confirmed complete isodisomy of chromosome 2 with detection of a homozygous frameshift likely pathogenic variant in *SPR* (p.Leu222CysfsTer4, chromosome 2p13.2; Fig. 2A ) and a homozygous missense variant of uncertain significance (VUS) in *ZNF142* (p.Arg823Gln, chromosome 2q35; Fig. 2B ). Biallelic pathogenic variants in *SPR* cause SRD, a dopa-responsive dystonia. Biallelic pathogenic variants in *ZNF142* cause NEDISHM.

Analysis of parental DNA indicated that neither variant was paternally inherited. Instead both were maternally inherited, compatible with maternal UPD2 in the patient ( Figs. 2A, B ). Paternity was confirmed by genome-wide rare allele analysis. A peripheral blood karyotype was not obtained because the family did not have health insurance.

The diagnosis of SRD was made and the patient was treated with levodopa. Follow-up shortly after showed dramatic improvement in her motor skills, but her expressive speech remained delayed with only a few syllables. Her receptive language, however, continued to be more advanced and she was able to follow multistep instructions.

A year later, the patient had almost normal gross motor skills. She could run, jump, and had nearly normal playground activities. Her fine motor skills remained delayed, but she was able to draw and color, and could feed herself using utensils. She was toilet trained and could dress herself, although she could not fasten buttons. She used single-syllable words and technology-assisted methods to communicate. Her receptive language remained advanced and she was bilingual. She interacted well with other children in school.

The diagnosis of NEDISHM remains uncertain because she does not have the abnormal movements or seizures described in affected individuals.

## Discussion of Case

Our patient has a clinical presentation consistent with features of both SRD and NEDISHM, disorders that have significant clinical overlap. This is the first reported case of UPD2 leading to homozygosity for a likely pathogenic variant in *SPR* and a VUS in *ZNF142* . The pathogenicity of both variants was interpreted according to 2015 American College of Genetics and Genomics (ACMGG) guidelines. 6

SRD is a rare, autosomal, recessive dopa-responsive dystonia caused by biallelic pathogenic variants in *SPR* , which encodes an aldo–keto reductase involved in the biosynthesis of BH _4_ . BH _4_ is a cofactor in the biosynthesis of neurotransmitters.

Our patient carried a homozygous 1-bp deletion in *SPR* , a gene with three exons. This change creates a premature stop codon in the last exon, which results in a truncated protein. Pathogenic variants have been reported in all three exons. 7 8 9 Two downstream nonsense variants (p.K230*; p.K251*) have previously been reported in patients with SRD. 8 10 11 12 Taking all evidence together, this 1-bp deletion is classified as a likely pathogenic variant (PVS1, PM2). 6 13

The clinical phenotype of SRD ranges from mild to severe motor and neurologic deficits. 14 Our patient's upward deviation of the eyes is likely to be oculogyric crisis, one of the major features of SRD present in more than 65% of affected individuals. 8 It is possible that oculogyric crises in our patient were mistaken for seizures given her normal EEG, although seizures may also occur in SRD. Our patient has other major features of SRD, including axial hypotonia and speech delay. Additional features of SRD which are also present in our patient include intellectual disability, brisk reflexes, and tremors when she was younger. This is the first report of UPD2 as a disease mechanism for SRD.

NEDISHM is a neurodevelopmental disorder caused by biallelic pathogenic variants in *ZNF142* . *ZNF142* is a zinc finger transcription factor expressed in all tissue types with high levels of expression in the cerebellum (GTEx database). A 2019 study by Khan et al identified seven affected individuals from four unrelated families with biallelic *ZNF142* mutations causing NEDISHM. 15 The clinical presentation of these individuals included cognitive impairment, speech deficits, motor impairment, tonic-clonic seizures, tremor, and dystonia. The homozygous missense variant in *ZNF142* found in our patient results in an arginine-to-glutamine substitution. In silico tools did not consistently support a deleterious effect of this change on the gene product (REVEL: 0.156); it has not previously been reported as disease-causing. The allele frequency of this variant is 18 in 1,613,950 alleles in gnomAD v4.0; 4 in 280,892 alleles in gnomAD v2.1. Based on the available evidence, it is classified as a VUS (ACMGG guideline: PM2 only). Given the significant overlap in phenotypic abnormalities between SRD and NEDISHM, we cannot draw a definitive conclusion about the *ZNF142* variant's contribution to our patient's phenotype.

A peripheral blood karyotype was not performed in this case. However, there was no indication of trisomy 2 mosaicism based on whole exome sequencing analysis. Thus, cryptic mosaicism for a trisomy 2 cell line as a contributor to the patient's phenotype is unlikely but cannot be excluded. Nonpaternity as an explanation for the molecular findings has been excluded by genome-wide rare allele analysis.

## Review of Published Reports of Uniparental Disomy of Chromosome 2

In addition to our case, we found 66 published reports of UPD2 for which clinical information is available. Table 1 adds 37 additional cases of UPD2 reported since Haudry et al's 2012 review, and also adds 12 additional cases of UPD2 published prior to 2012 and not cited by Haudry et al. 16 In addition, Table 1 includes reports of phenotypically normal individuals in whom UPD2 was incidentally found via paternity testing, testing for a familial disease, or single nucleotide polymorphism chromosome microarray of amniocyte DNA performed for maternal age.

**Table 1:** Reported cases of chromosome 2 uniparental disomy

Year	Reference	Parental origin	Phenotype	Age at assessment of normal phenotype	Gene	Segmental or complete UPD	Type of UPD	Number of patients
1995	Harrison et al 17	Maternal	IUGR, oligohydramnios; normal development	31 months	Unknown	Complete	Heterodisomy with segmental isodisomy	1
1995	Bernard et al	Maternal	(Abstract only; could not locate paper)	–	(Abstract only)	(Abstract only)	(Abstract only)	1
1996	Bernasconi et al 23	Maternal	Normal healthy female with recurrent pregnancy loss due to isochromosome 2p and isochromosome 2q (46,XX,i[2][p10],i[2][q10])	36 years	None	Complete	Isodisomy	1
1996	Webb et al 19	Maternal	SGA, oligohydramnios, echogenic kidneys, pyloric stenosis; normal development	5 years	Unknown	Complete	Heterodisomy with segmental isodisomy	1
1997	Hansen et al 20	Maternal	IUGR, oligohydramnios, and hypospadias with chordee; neonatal death	–	Unknown	Not reported	Heterodisomy	1
1997	Shaffer et al 22	Maternal	IUGR and oligohydramnios with maternal isochromosome 2p and maternal isochrome 2q (46,XY,i[2][p10],i[2][q10])	–	Unknown	Complete	Isodisomy	1
2000	Chávez et al a 38	Paternal	Pseudohermaphroditism	–	*SRD5A2*	Complete	Isodisomy	1
2000	Heide et al 25	Maternal	Normal phenotype b	3 years	N/A	Complete	Heterodisomy with segmental isodisomy	1
2001	Bakker et al a 39	Maternal	Severe congenital hypothyroidism	–	*TPO*	Segmental	Isodisomy	1
2001	Stratakis et al a 40	Maternal	Normal phenotype b	22 years	N/A	Segmental	Isodisomy	1
2001	Wolstenholme et al 18	Maternal	SGA, oligohydramnios; normal development	6 months	N/A	Complete	Heterodisomy	1
2001	Albrecht et al 41	Mixed	Normal healthy female with recurrent pregnancy loss due to uniparental isodisomy for paternal 2p and maternal 2q	36 years	N/A	Segmental	Maternal 2q, paternal 2p	1
2002	Spiekerkoetter et al a 42	Maternal	Mitochondrial trifunctional protein deficiency	–	*HADHA*	Unknown	Unknown	2
2002	Thompson et al a 43	Paternal	Retinal dystrophy	–	*MERTK*	Complete	Isodisomy	1
2003	Latronico et al a 44	Maternal	Familial male-limited precocious puberty	–	*LHR*	Complete	Isodisomy	1
2005	Petit et al a 45	Paternal	Crigler–Najjar syndrome type I	–	*UGT1A1*	Complete	Isodisomy	1
2005	Chevalier-Porst et al a 46	Maternal	Primary hyperoxaluria type 1	–	*AGXT*	Segmental	Isodisomy	1
2007	Baumer et al 47	Mixed	Normal healthy male with female partner with recurrent pregnancy loss due to uniparental isodisomy for maternal 2q and paternal 2p	Adulthood, age unspecified	N/A	Segmental	Maternal 2q, paternal 2p	1
2008	Kantarci et al a 48	Paternal	Donnai–Barrow syndrome	–	*LRP2*	Complete	Isodisomy	1
2009	López-Garrido et al a 49	Paternal	Primary congenital glaucoma	–	*CYP1B1*	Complete	Isodisomy	1
2009	Herzfeld et al a 50	Maternal	Infantile-onset ascending spastic paralysis	–	*ALS2*	Complete	Heterodisomy with segmental isodisomy	1
2009	Hamvas et al a 51	Maternal	Surfactant protein B deficiency	–	*SFTPB*	Complete	Heterodisomy with segmental isodisomy	1
2009	Castiglia et al 52	Paternal	Harlequin ichthyosis	–	*ABCA12*	Complete	Isodisomy	1
2009	Keller et al 24	Paternal	Normal phenotype	22 years	N/A	Complete	Isodisomy	1
2009	Talseth-Palmer et al a 34	Paternal	Syndromic intellectual disability	–	Unknown	Complete	Isodisomy	1
2010	Baskin et al a 53	Paternal	Long-chain 3-hydroxyacyl-CoA dehydrogenase deficiency	–	*HADHA*	Complete	Isodisomy	1
2011	Douglas et al a 54	Paternal	Hepatocerebral mitochondrial DNA depletion syndrome	–	*DGUOK*	Complete	Isodisomy	1
2012	Haudry et al a 16	Maternal	Hepatocerebral mitochondrial DNA depletion syndrome	–	*DGUOK*	Complete	Heterodisomy with segmental isodisomy	1
2012	Giovannoni et al a 55	Paternal	Progressive familial intrahepatic cholestasis type II	–	*ABCB11*	Segmental	Isodisomy	1
2013	Carmichael et al 36	Maternal	Retinal dystrophy with complex phenotype	–	17 candidates including *FAM161A, NAT8, PLA2R1, NPHP4, ARL6IP6*	Complete	Isodisomy	1
2013	Ou et al 26	Paternal	Normal phenotype b	Not reported	N/A	Complete	Isodisomy	1
2014	Quintana et al 35	Maternal	Alobar holoprosencephaly	–	Unknown	Not reported	Isodisomy	1
2015	Meijer et al 56	Maternal	Rhabdomyolysis	–	*LPIN1*	Complete	Isodisomy	1
2016	Yu et al 57	Maternal	Obesity and developmental delay	–	*GPBAR1, CAPN10*	Complete	Isodisomy	1
2016	Dasi et al 58	Paternal	Vitamin K-dependent coagulant factor deficiency	–	*GGCX*	Complete	Isodisomy	1
2018	Shen et al 59	Paternal	Lethal multiple pterygium syndrome	–	*CHRND*	Complete	Isodisomy	1
2019	Zhang et al 27	Paternal	Normal phenotype c	18 years	N/A	Complete	Isodisomy	1
2019	Smigiel et al 60	Maternal	Growth retardation, alopecia, pseudoanodontia, and optic atrophy syndrome	–	*ANTXR1*	Complete	Isodisomy	1
2019	Souzeau et al 61	Paternal	Primary congenital glaucoma	–	*CYP1B1*	Complete	Isodisomy	1
2019	Chen et al 28	Paternal	Normal phenotype c	Not reported	N/A	Complete	Isodisomy	1
2019	Guzmán-Alberto et al 62	Maternal	Not assessed	–	N/A	Complete	Isodisomy	1
2019	Shyla et al 63	Maternal	Miller–Dieker syndrome (clinical diagnosis; negative molecular diagnosis)	–	N/A	Suspected complete	Isodisomy	1
2019	Panasiti et al 64	Paternal	Progressive familial intrahepatic cholestasis type 2	–	*ABCB11*	Not reported	Isodisomy	1
2020	Zhang et al 65	Maternal	Congenital myasthenic syndrome 22	–	*PREPL*	Complete	Isodisomy	1
2020	Higgins et al 66	Maternal	Epidermolysis bullosa	–	*ITAG6*	Complete	Isodisomy	1
2020	Horga et al 67	Maternal	*MRPL44* -related disease	–	*MRLP44*	Complete	Isodisomy	1
2020	Doniec et al 29	Maternal	Normal phenotype c	Not reported	N/A	Complete	Heterodisomy with segmental isodisomy	1
2020	Xia et al 68	Paternal	Mitochondrial DNA depletion syndrome	–	*DGUOK*	Complete	Isodisomy	1
2020	Takenouchi et al 69	Paternal	Protein C deficiency	–	*PROC*	Complete	Isodisomy	1
2020	Song et al 30	Not reported	Normal phenotype	18 months	N/A	Complete	Isodisomy	1
2020	Szelinger et al 70	Maternal	Congenital myasthenic syndrome	–	*GFPT1*	Not reported	Isodisomy	1
2020	Shchagina et al 71	Maternal	Congenital myasthenic syndrome-22	–	*PREPL*	Suspected complete	Isodisomy	1
2020	Sezer et al 72	Paternal	Warburg Micro Syndrome 1	–	*RAB3GAP1*	Complete	Heterodisomy with segmental isodisomy	1
2020	Prasov et al 73	Paternal	Jalili syndrome	–	*CNNM4*	Not reported	Isodisomy	1
2021	Kohl et al 74	Paternal	Achromatopsia	–	*CNGA3*	Suspected complete	Isodisomy	1
2021	Schüle et al 75	Maternal	Catel–Manzke Syndrome/VCRL Syndrome	–	*KYNU*	Complete	Isodisomy	1
2021	Knapp et al 76	Paternal	Crigler–Najjar syndrome type I and long-chain 3-hydroxyacyl-CoA dehydrogenase deficiency	–	*HADHA, UGT1A1*	Complete	Isodisomy	1
2021	Hara-Isono et al 77	Paternal	Schimke immuno-osseous dysplasia	–	*SMARCAL1*	Complete	Isodisomy	1
2021	Tao et al 78	Maternal	Infantile hypotonia with psychomotor retardation and characteristic facies 2	–	*UNC80*	Complete	Isodisomy	1
2022	Li et al 79	Paternal	Dysferlinopathy	–	*DYSF*	Complete	Isodisomy	1
2022	Lopour et al 80	Maternal	Alström syndrome	–	*ALMS1*	Complete	Heterodisomy with segmental isodisomy	1
2022	Molloy et al 81	Paternal	Dystonia–parkinsonism phenotype		*PRKRA*	Complete	Isodisomy	1
2022	Molloy et al 81	Paternal	Lymphopenia, specific pneumococcal antibody deficiency	–	Unknown	Complete	Isodisomy	1
2023	Nishimura-Kinoshita et al 82	Maternal	Hyperphosphatemic familial tumoral calcinosis	–	*GALNT3*	Complete	Isodisomy	1
2023	Jain et al 83	Paternal	Congenital hypothyroidism	–	*TPO*	Complete	Isodisomy	1
2023	Chen et al 84	Maternal	Normal phenotype	12 months	N/A	Not reported	Heterodisomy	1
2023	Current report	Maternal	Sepiapterin reductase deficiency and neurodevelopmental disorder with impaired speech and hyperkinetic movements	–	*ZNF142, SPR*	Complete	Isodisomy	1

Abbreviations: IUGR, intrauterine growth restriction; N/A, not applicable; SGA, small for gestational age; UPD, uniparental disomy.

Adapted from Haudry et al. 16

a Papers included in Haudry et al review.

b Incidental finding during testing for a familial disease.

c Identified via paternity testing.

There are a number of reasons why an abnormal phenotype may be present in the setting of UPD2. The mechanism most commonly reported is exposure of an autosomal recessive disorder, as illustrated by our case. In our review of the literature, maternal or paternal uniparental isodisomy of chromosome 2 was identified as the cause of an autosomal recessive disorder in 42 cases ( Fig. 1 ). In addition, the presence of UPD2 also raises the possibility of cryptic mosaicism for a trisomy 2 (or, less likely, monosomy 2) cell line in the embryo. There also may be adverse effects on development due to placental dysfunction caused by confined placental mosaicism for a trisomy 2 cell line or a cell line with UPD2. There are no reports suggesting that chromosome 2 contains imprinted genes that are a cause of adverse outcomes in the setting of UPD2.

There are five reports of unrelated infants with UPD2 whose mothers had presumptive confined placental mosaicism for trisomy 2. These pregnancies were complicated by severe IUGR and oligohydramnios. Postnatally, the infants were found to have normal karyotypes as determined by analysis of cord blood, peripheral blood cells, and/or skin fibroblasts. One case reported growth below the 10th centile at 14 months but otherwise normal motor and intellectual development. 17 Another case reported weight 3.5 standard deviations (SD) below the mean at 6 months but otherwise normal development without dysmorphic features. 18 Webb et al described a patient whose postnatal course was complicated by renal failure, congenital pyloric stenosis, and hiatal hernia requiring multiple surgeries and gastrostomy tube placements. 19 At 5 years, the child was nondysmorphic with normal development and weight had improved from less than 5 SD below the mean at 8 months to 25th to 50th percentile. 18 19 One case resulted in neonatal death due to complications of severe IUGR. 20 Another case had normal development at 1-year follow-up. 21 Long-term follow-up into adulthood is not available for the infants whose gestations were complicated by presumptive confined placental mosaicism for a trisomic cell line. This is a significant limitation to counseling about the potential for long-term adverse effects on development due to the effects of UPD2, placental mosaicism for a trisomy 2 cell line, and the possibility of cryptic chromosomal mosaicism in a child.

A case involving UPD2 caused by the presence of two maternal isochromosomes (46,XY,i[2][p10];i[2][10]) had some clinical features similar to the cases of presumed confined placental mosaicism, including IUGR and oligohydramnios. However, this patient also had hypospadias, preauricular ear pits, pectus carinatum, and fifth-finger clinodactyly. At 8 years of age, height remained below 2 SD of the mean. No information about neurodevelopment was provided. 22 Whole exome sequencing and chromosome microarray were not performed.

Eight cases of phenotypically normal individuals who were incidentally found to have complete UPD2 have been reported. These cases were identified by paternity testing, genetic analysis performed as part of a family or research study, and SNP array performed after amniocentesis for a nonfetal indication. Two of these cases had complete maternal UPD2; four had complete paternal UPD2; one had complete maternal isodisomy resulting from two maternal isochromosomes i(2q) and i(2p). 23 24 25 26 27 28 29 Parental origin was not reported in one case. 30

The ages at which individuals with UPD2 and a normal phenotype was reported ranged from 18 months to 36 years. Three cases did not report age. In addition, four cases of UPD2 associated with presumptive confined placental mosaicism with fetal growth restriction and oligohydramnios have been reported with normal neurological development at ages ranging from 6 months to 5 years. 17 18 19 21 Reports of normal phenotypes associated with both maternal and paternal UPD2 provide support for the conclusion that genes located on chromosome 2 are not imprinted.

We note that a recent report by Tan et al speculated that imprinting might account for the severe IUGR noted in two fetuses with UPD2, one of whom died in utero. 31 However, this report did not include information about cytogenetic studies of the placentas and thus could not exclude the possibility of confined placental mosaicism for trisomy 2 cells for their cases. It is well established that placental mosaicism for trisomy 2 cells, which can be associated with fetal UPD2 due to a trisomy rescue event, can result in placental dysfunction leading to IUGR. 32 33 Placental mosaicism for trisomy 2 cells is a far more likely explanation for the findings of Tan et al, given the strong evidence that genes located on chromosome 2 are not subject to imprinting.

Among most reported cases of autosomal recessive disorders caused by UPD, the phenotypes could be fully explained by the expression of biallelic pathogenic variants, providing indirect evidence against imprinted genes on chromosome 2. Three papers report cases of UPD2 with an abnormal phenotype including multiple congenital anomalies without a known monogenic cause, although whole exome sequencing was not performed in two of the cases. 34 35 Carmichael et al reported a case of maternal isodisomy of chromosome 2 in association with a complex phenotype including skeletal and renal dysplasia, immune deﬁciencies, growth failure, retinal degeneration and ovarian insufﬁciency. 36 The patient underwent whole exome sequencing which detected 18 rare homozygous variants on chromosome 2 and another 5 genes on other chromosomes with compound heterozygous possibly pathogenic variants. No definitively causal pathogenic variant(s) was identified.

## Factors Underlying Uniparental Disomy of Chromosome 2

Including our case report, 53 cases of complete UPD2 have been identified. Of these cases, 27 were maternal and 26 were paternal. Similarly, Haudry et al in 2012 found that cases of maternal and paternal UPD2 occurred with equal frequency. However, a 2021 review of UPD across all chromosomes using 32,067 whole exome parent–child trios referred for a diverse set of indications including neurodevelopmental abnormalities found complete maternal UPD to occur significantly more frequently than complete paternal UPD (69 maternal UPD cases and 30 paternal UPD cases). 4 The lower prevalence of complete maternal UPD cases in our and Haudry et al's literature reviews could be explained by the underreporting of maternal heterodisomy cases because they are not associated with autosomal recessive conditions.

This hypothesis is supported by our finding that most cases of heterodisomy for UPD2 were maternal in origin (11 out of 12). Scuffins et al also found that, among cases of heterodisomy and mixed UPD for all chromosomes (defined as complete heterodisomy with segmental isodisomy), the parent of origin was maternal in 55/60 events (91.6%). 4 In a 2004 Austrian study, Kotzot reported 145 pregnancies complicated by UPD for any chromosome; among the 80 cases of heterodisomy, the parent of origin was maternal in 74/80 (92.5%). 37 The increased prevalence of maternal heterodisomy provides support for Kotzot's speculation that maternal meiosis I nondisjunction, rather than paternal meiosis I nondisjunction, is the major contributor to uniparental heterodisomy.

Of the 43 cases of complete or suspected complete chromosome 2 isodisomy listed in Table 1 , only 18 of 43 (42%) were maternal in origin. Similarly, in cases of complete isodisomy, Scuffins et al found that the parent of origin was maternal in 14/39 events (35.9%). 4 A high proportion of paternal UPD cases are isodisomic. Almost 80% (28/34) of cases of paternal UPD in Kotzot's large series were associated with isodisomy; in contrast, only one-third (37/111) of maternal UPD in the Kotzot study were associated with isodisomy. In the Scuffins study, of the 27 cases of complete paternal UPD, 92.6% (25 cases) were isodisomy. 4 The high proportion of paternal isodisomy provides evidence that in the setting of normal parental karyotypes, paternal UPD more commonly arises due to either meiosis II nondisjunction or postzygotic monosomy rescue, both of which would result in isodisomy. Support for monosomy rescue being a major contributor to paternal isodisomy is the lower frequency of aneuploidy in sperm than in ova. 37

The risk of meiotic nondisjunction is directly correlated with maternal age, and therefore maternal age is a risk factor for fetal UPD. Scuffins et al found that the average maternal age of maternal UPD cases (37.4 years) was significantly higher than maternal age of cases without UPD (30.3 years [ *p* = 0.000001]). 4 In 2004, Kotzot reported 111 pregnancies complicated by UPD for any chromosome (excluding chromosome 15) in which maternal age was reported, and 34 pregnancies complicated by UPD (excluding chromosome 15) in which paternal age was reported. The mean maternal age for 74 cases of maternal heterodisomy was 34.8 years, which is significantly older than the mean maternal age of pregnant women in the general Austrian population of 30 years ( *p* < 0.0001). In contrast, the mean maternal age of 29 years for the 37 cases of maternal isodisomy was not significantly different than the mean maternal age in the general Austrian population. 37 These data suggest that meiosis I errors, which can lead to heterodisomy, occur with greater frequency with advancing maternal age whereas meiosis II errors, which lead to isodisomy do not appear to be influenced by maternal age.

The mean paternal age in the 28 cases of paternal isodisomy in the Austrian study was 31.2 years, which is the same as the mean paternal age in the Austrian population. 37 Due to the small number of cases of paternal heterodisomy, the mean paternal age was not reported. We could not evaluate the association of parental age with UPD2 in our literature review because parental ages were reported for only 13 cases. Including parental age in future case reports about UPD is strongly encouraged and would provide more insight into the mechanisms by which UPD occurs.

The presence of two or more autosomal recessive disorders with loci on the same chromosome in an individual is one of several indications to test for UPD. In our case, homozygosity at two alleles on different arms of chromosome 2 and the results of SNP analysis strongly suggest complete maternal uniparental isodisomy of chromosome 2. Current information suggests that chromosome 2 does not contain imprinted genes. The Scuffins whole exome trio analysis also did not find evidence of imprinting disorders on chromosome 2. 4 Additional reports of UPD2 will continue to provide information about this possibility as well as information about the pathogenicity of rare gene variants located on chromosome 2. From the genetic counseling perspective, diagnosing a child with an autosomal recessive disorder caused by UPD reduces the risk of recurrence of another affected child from 25% to close to the general population risk in parents who have normal karyotypes.

## References

[1] Engel E A new genetic concept: uniparental disomy and its potential effect, isodisomy Am J Med Genet 1980 6 02 137 143 7192492 10.1002/ajmg.1320060207

[2] ACMG Laboratory Quality Assurance Committee Del Gaudio D Shinawi M Astbury C Tayeh M K Deak K L Raca G Diagnostic testing for uniparental disomy: a points to consider statement from the American College of Medical Genetics and Genomics (ACMG) Genet Med 2020 22 07 1133 1141 32296163 10.1038/s41436-020-0782-9

[3] Robinson W P Mechanisms leading to uniparental disomy and their clinical consequences BioEssays 2000 22 05 452 459 10797485 10.1002/(SICI)1521-1878(200005)22:5<452::AID-BIES7>3.0.CO;2-K

[4] Scuffins J Keller-Ramey J Dyer L Uniparental disomy in a population of 32,067 clinical exome trios Genet Med 2021 23 06 1101 1107 33495530 10.1038/s41436-020-01092-8 PMC8187148

[5] Silverstein S Lerer I Sagi M Frumkin A Ben-Neriah Z Abeliovich D Uniparental disomy in fetuses diagnosed with balanced Robertsonian translocations: risk estimate Prenat Diagn 2002 22 08 649 651 12210570 10.1002/pd.370

[6] ACMG Laboratory Quality Assurance Committee Richards S Aziz N Bale S Standards and guidelines for the interpretation of sequence variants: a joint consensus recommendation of the American College of Medical Genetics and Genomics and the Association for Molecular Pathology Genet Med 2015 17 05 405 424 25741868 10.1038/gim.2015.30 PMC4544753

[7] Blau N PKU and BH4: Advances in Phenylketonuria and TetrahydrobiopterinSPS Publ.;2006

[8] Friedman J Roze E Abdenur J E Sepiapterin reductase deficiency: a treatable mimic of cerebral palsy Ann Neurol 2012 71 04 520 530 22522443 10.1002/ana.22685

[9] Koht J Rengmark A Opladen T Clinical and genetic studies in a family with a novel mutation in the sepiapterin reductase gene Acta Neurol Scand Suppl 2014 198 7 12 24588500 10.1111/ane.12230

[10] Echenne B Roubertie A Assmann B Sepiapterin reductase deficiency: clinical presentation and evaluation of long-term therapy Pediatr Neurol 2006 35 05 308 313 17074599 10.1016/j.pediatrneurol.2006.05.006

[11] Arrabal L Teresa L Sánchez-Alcudia R Genotype-phenotype correlations in sepiapterin reductase deficiency. A splicing defect accounts for a new phenotypic variant Neurogenetics 2011 12 03 183 191 21431957 10.1007/s10048-011-0279-4

[12] Leuzzi V Carducci C Tolve M Giannini M T Angeloni A Carducci C Very early pattern of movement disorders in sepiapterin reductase deficiency Neurology 2013 81 24 2141 2142 24212389 10.1212/01.wnl.0000437299.51312.5f

[13] ClinGen Sequence Variant Interpretation Working Group (ClinGen SVI) Abou Tayoun A N Pesaran T DiStefano M T Recommendations for interpreting the loss of function PVS1 ACMG/AMP variant criterion Hum Mutat 2018 39 11 1517 1524 30192042 10.1002/humu.23626 PMC6185798

[14] Friedman J Sepiapterin Reductase Deficiency University of Washington; 2015 26131547

[15] Khan K Zech M Morgan A T Recessive variants in ZNF142 cause a complex neurodevelopmental disorder with intellectual disability, speech impairment, seizures, and dystonia Genet Med 2019 21 11 2532 2542 31036918 10.1038/s41436-019-0523-0 PMC6821592

[16] Haudry C de Lonlay P Malan V Maternal uniparental disomy of chromosome 2 in a patient with a DGUOK mutation associated with hepatocerebral mitochondrial DNA depletion syndrome Mol Genet Metab 2012 107 04 700 704 23141463 10.1016/j.ymgme.2012.10.008

[17] Harrison K Eisenger K Anyane-Yeboa K Brown S Maternal uniparental disomy of chromosome 2 in a baby with trisomy 2 mosaicism in amniotic fluid culture Am J Med Genet 1995 58 02 147 151 8533806 10.1002/ajmg.1320580211

[18] Wolstenholme J White I Sturgiss S Carter J Plant N Goodship J A Maternal uniparental heterodisomy for chromosome 2: detection through 'atypical' maternal AFP/hCG levels, with an update on a previous case Prenat Diagn 2001 21 10 813 817 11746120 10.1002/pd.143

[19] Webb A L Sturgiss S Warwicker P Robson S C Goodship J A Wolstenholme J Maternal uniparental disomy for chromosome 2 in association with confined placental mosaicism for trisomy 2 and severe intrauterine growth retardation Prenat Diagn 1996 16 10 958 962 8938070 10.1002/(SICI)1097-0223(199610)16:10<958::AID-PD971>3.0.CO;2-U

[20] Hansen W F Bernard L E Langlois S Maternal uniparental disomy of chromosome 2 and confined placental mosaicism for trisomy 2 in a fetus with intrauterine growth restriction, hypospadias, and oligohydramnios Prenat Diagn 1997 17 05 443 450 9178319 10.1002/(sici)1097-0223(199705)17:5<443::aid-pd82>3.0.co;2-2

[21] Chen C-P Wu F T Chern S R Low-level mosaic trisomy 2 at amniocentesis in a pregnancy associated with positive NIPT and CVS results for trisomy 2, maternal uniparental disomy 2, perinatal progressive decrease of the aneuploid cell line, cytogenetic discrepancy between cultured amniocytes and uncultured amniocytes, intrauterine growth restriction and a favorable fetal outcome Taiwan J Obstet Gynecol 2023 62 04 571 576 37407197 10.1016/j.tjog.2023.05.002

[22] Shaffer L G McCaskill C Egli C A Baker J C Johnston K M Is there an abnormal phenotype associated with maternal isodisomy for chromosome 2 in the presence of two isochromosomes? Am J Hum Genet 1997 61 02 461 462 9311755 10.1016/S0002-9297(07)64076-2 PMC1715890

[23] Bernasconi F Karagüzel A Celep F Normal phenotype with maternal isodisomy in a female with two isochromosomes: i(2p) and i(2q) Am J Hum Genet 1996 59 05 1114 1118 8900241 PMC1914849

[24] Keller M C McRae A F McGaughran J M Visscher P M Martin N G Montgomery G W Non-pathological paternal isodisomy of chromosome 2 detected from a genome-wide SNP scan Am J Med Genet A 2009 149A 08 1823 1826 19610117 10.1002/ajmg.a.32973

[25] Heide E Heide K G Rodewald A Maternal uniparental disomy (UPD) for chromosome 2 discovered by exclusion of paternity Am J Med Genet 2000 92 04 260 263 10842292

[26] Ou X Liu C Chen S Complete paternal uniparental isodisomy for Chromosome 2 revealed in a parentage testing case Transfusion 2013 53 06 1266 1269 22924962 10.1111/j.1537-2995.2012.03863.x

[27] Zhang X Ding Z He R Qi J Zhang Z Cui B Complete paternal uniparental disomy of chromosome 2 in an Asian female identified by short tandem repeats and whole genome sequencing Cytogenet Genome Res 2019 157 04 197 202 30991391 10.1159/000499893

[28] Chen M Jiang J Li C Non-pathological complete paternal uniparental isodisomy of chromosome 2 revealed in a maternity testing case Int J Legal Med 2019 133 04 993 997 29802460 10.1007/s00414-018-1857-x

[29] Doniec A Łuczak W Wróbel M Confirmation of paternity despite three genetic incompatibilities at chromosome 2 Genes (Basel) 2021 12 01 62 33406744 10.3390/genes12010062 PMC7824413

[30] Song J Zhu L Zhang C Wu Y Wang B A rare case of complete uniparental isodisomy of chromosome 2 with no phenotypic abnormalities Taiwan J Obstet Gynecol 2021 60 02 378 379 33678350 10.1016/j.tjog.2021.01.024

[31] Tan X Liu B Yan T Prenatal diagnosis of paternal uniparental disomy for chromosome 2 in two fetuses with intrauterine growth restriction Mol Cytogenet 2023 16 01 20 37612666 10.1186/s13039-023-00647-z PMC10464012

[32] Sifakis S Staboulidou I Maiz N Velissariou V Nicolaides K H Outcome of pregnancies with trisomy 2 cells in chorionic villi Prenat Diagn 2010 30 04 329 332 20120006 10.1002/pd.2457

[33] Talantova O E Koltsova A S Tikhonov A V Prenatal detection of trisomy 2: considerations for genetic counseling and testing Genes (Basel) 2023 14 04 913 37107671 10.3390/genes14040913 PMC10138005

[34] Talseth-Palmer B A Bowden N A Meldrum C A 1q44 deletion, paternal UPD of chromosome 2 and a deletion due to a complex translocation detected in children with abnormal phenotypes using new SNP array technology Cytogenet Genome Res 2009 124 01 94 101 19372674 10.1159/000200093

[35] Quintana A Garabedian M J Wallerstein R J Antenatal detection of maternal unipartental disomy of chromosome 2 in a fetus with non-chromosomal, non-syndromic alobar holoprosencephaly Am J Med Genet A 2014 164A 01 276 278 24243683 10.1002/ajmg.a.36204

[36] Carmichael H Shen Y Nguyen T T Hirschhorn J N Dauber A Whole exome sequencing in a patient with uniparental disomy of chromosome 2 and a complex phenotype Clin Genet 2013 84 03 213 222 23167750 10.1111/cge.12064 PMC3996682

[37] Kotzot D Advanced parental age in maternal uniparental disomy (UPD): implications for the mechanism of formation Eur J Hum Genet 2004 12 05 343 346 14747835 10.1038/sj.ejhg.5201158

[38] Chávez B Valdez E Vilchis F Uniparental disomy in steroid 5alpha-reductase 2 deficiency J Clin Endocrinol Metab 2000 85 09 3147 3150 10999800 10.1210/jcem.85.9.6786

[39] Bakker B Bikker H Vulsma T de Randamie J S Wiedijk B M De Vijlder J J Two decades of screening for congenital hypothyroidism in The Netherlands: TPO gene mutations in total iodide organification defects (an update) J Clin Endocrinol Metab 2000 85 10 3708 3712 11061528 10.1210/jcem.85.10.6878

[40] Stratakis C A Taymans S E Schteingart D Haddad B R Segmental uniparental isodisomy (UPD) for 2p16 without clinical symptoms: implications for UPD and other genetic studies of chromosome 2 J Med Genet 2001 38 02 106 109 11288708 10.1136/jmg.38.2.106 PMC1734803

[41] Albrecht B Mergenthaler S Eggermann K Zerres K Passarge E Eggermann T Uniparental isodisomy for paternal 2p and maternal 2q in a phenotypically normal female with two isochromosomes, i(2p) and i(2q) J Med Genet 2001 38 03 214 11303520 10.1136/jmg.38.3.214 PMC1734826

[42] Spiekerkoetter U Eeds A Yue Z Haines J Strauss A W Summar M Uniparental disomy of chromosome 2 resulting in lethal trifunctional protein deficiency due to homozygous alpha-subunit mutations Hum Mutat 2002 20 06 447 451 12442268 10.1002/humu.10142

[43] Thompson D A McHenry C L Li Y Retinal dystrophy due to paternal isodisomy for chromosome 1 or chromosome 2, with homoallelism for mutations in RPE65 or MERTK, respectively Am J Hum Genet 2002 70 01 224 229 11727200 10.1086/338455 PMC384890

[44] Latronico A C Billerbeck A E Pinto E M Brazil D'Alva C Arnhold I J Mendonca B B Maternal isodisomy causing homozygosity for a dominant activating mutation of the luteinizing hormone receptor gene in a boy with familial male-limited precocious puberty Clin Endocrinol (Oxf) 2003 59 04 533 534 14510919 10.1046/j.1365-2265.2003.01810.x

[45] Petit F M Gajdos V Parisot F Paternal isodisomy for chromosome 2 as the cause of Crigler-Najjar type I syndrome Eur J Hum Genet 2005 13 03 278 282 15586176 10.1038/sj.ejhg.5201342

[46] Chevalier-Porst F Rolland M O Cochat P Bozon D Maternal isodisomy of the telomeric end of chromosome 2 is responsible for a case of primary hyperoxaluria type 1 Am J Med Genet A 2005 132A 01 80 83 15580638 10.1002/ajmg.a.30375

[47] Baumer A Basaran S Taralczak M Initial maternal meiotic I error leading to the formation of a maternal i(2q) and a paternal i(2p) in a healthy male Cytogenet Genome Res 2007 118 01 38 41 17901698 10.1159/000106439

[48] Kantarci S Ragge N K Thomas N S Donnai-Barrow syndrome (DBS/FOAR) in a child with a homozygous LRP2 mutation due to complete chromosome 2 paternal isodisomy Am J Med Genet A 2008 146A 14 1842 1847 18553518 10.1002/ajmg.a.32381 PMC2891749

[49] López-Garrido M P Campos-Mollo E Harto M A Escribano J Primary congenital glaucoma caused by the homozygous F261L CYP1B1 mutation and paternal isodisomy of chromosome 2 Clin Genet 2009 76 06 552 557 19807744 10.1111/j.1399-0004.2009.01242.x

[50] Herzfeld T Wolf N Winter P Hackstein H Vater D Müller U Maternal uniparental heterodisomy with partial isodisomy of a chromosome 2 carrying a splice acceptor site mutation (IVS9-2A>T) in ALS2 causes infantile-onset ascending spastic paralysis (IAHSP) Neurogenetics 2009 10 01 59 64 18810511 10.1007/s10048-008-0148-y

[51] Hamvas A Nogee L M Wegner D J Inherited surfactant deficiency caused by uniparental disomy of rare mutations in the surfactant protein-B and ATP binding cassette, subfamily a, member 3 genes J Pediatr 2009 155 06 854 8590 19647838 10.1016/j.jpeds.2009.06.006 PMC2794197

[52] Castiglia D Castori M Pisaneschi E Trisomic rescue causing reduction to homozygosity for a novel ABCA12 mutation in harlequin ichthyosis Clin Genet 2009 76 04 392 397 19664001 10.1111/j.1399-0004.2009.01198.x

[53] Baskin B Geraghty M Ray P N Paternal isodisomy of chromosome 2 as a cause of long chain 3-hydroxyacyl-CoA dehydrogenase (LCHAD) deficiency Am J Med Genet A 2010 152A 07 1808 1811 20583174 10.1002/ajmg.a.33462

[54] Douglas G V Wiszniewska J Lipson M H Detection of uniparental isodisomy in autosomal recessive mitochondrial DNA depletion syndrome by high-density SNP array analysis J Hum Genet 2011 56 12 834 839 22011815 10.1038/jhg.2011.112 PMC7512120

[55] Giovannoni I Terracciano A Gennari F David E Francalanci P Santorelli F M Paternal isodisomy of chromosome 2 in a child with bile salt export pump deficiency Hepatol Res 2012 42 03 327 331 22364601 10.1111/j.1872-034X.2011.00925.x

[56] Meijer I A Sasarman F Maftei C *LPIN1* deficiency with severe recurrent rhabdomyolysis and persistent elevation of creatine kinase levels due to chromosome 2 maternal isodisomy Mol Genet Metab Rep 2015 5 85 88 28649549 10.1016/j.ymgmr.2015.10.010 PMC5471397

[57] Yu T Li J Li N Obesity and developmental delay in a patient with uniparental disomy of chromosome 2 Int J Obes 2016 40 12 1935 1941 10.1038/ijo.2016.160 27654142

[58] Dasi M A Gonzalez-Conejero R Izquierdo S Uniparental disomy causes deficiencies of vitamin K-dependent proteins J Thromb Haemost 2016 14 12 2410 2418 27681307 10.1111/jth.13517

[59] Shen W Young B A Bosworth M Wright K E Lamb A N Ji Y Prenatal detection of uniparental disomy of chromosome 2 carrying a CHRND pathogenic variant that causes lethal multiple pterygium syndrome Clin Genet 2018 93 06 1248 1249 29399782 10.1111/cge.13164

[60] Smigiel R Rozensztrauch A Walczak A Changing facial features in a child with GAPO syndrome caused by novel mutation in the ANTXR1 gene and uniparental disomy of chromosome 2 Clin Dysmorphol 2019 28 04 211 214 31425299 10.1097/MCD.0000000000000292

[61] Souzeau E Dubowsky A Ruddle J B Craig J E Primary congenital glaucoma due to paternal uniparental isodisomy of chromosome 2 and CYP1B1 deletion Mol Genet Genomic Med 2019 7 08 e774 31251480 10.1002/mgg3.774 PMC6687653

[62] Guzmán-Alberto J C Martínez-Cortes G Rangel-Villalobos H Inference of maternal uniparental disomy of the entire chromosome 2 from a paternity test Int J Legal Med 2019 133 01 71 75 29511852 10.1007/s00414-018-1811-y

[63] Shyla A Two loci 'exclusion' of true paternity is due to genetic disorder in a child Forensic Sci Int Genet Suppl Ser 2019 7 01 3 4

[64] Panasiti I Briuglia S Costa S Caminiti L Comorbidity between progressive familial intrahepatic cholestasis and atopic dermatitis in a 19-month-old child BMJ Case Rep 2019 12 10 e230152 10.1136/bcr-2019-230152 PMC6803136 31630127

[65] Zhang P Wu B Lu Y First maternal uniparental disomy for chromosome 2 with PREPL novel frameshift mutation of congenital myasthenic syndrome 22 in an infant Mol Genet Genomic Med 2020 8 03 e1144 31985178 10.1002/mgg3.1144 PMC7057094

[66] Higgins R Jensen A N Wachstein J Uniparental disomy of chromosome 2 unmasks new ITGA6 recessive mutation and results in a lethal junctional epidermolysis bullosa in a newborn Acta Derm Venereol 2020 100 01 adv00041 31502654 10.2340/00015555-3313 PMC9128983

[67] Horga A Manole A Mitchell A L Uniparental isodisomy of chromosome 2 causing MRPL44-related multisystem mitochondrial disease Mol Biol Rep 2021 48 03 2093 2104 33742325 10.1007/s11033-021-06188-1

[68] Xia J K Bai Z X Zhao X C Meng J J Chen C Kong X D Mitochondrial DNA depletion syndrome in a newborn with jaundice caused by DGUOK mutation and complete uniparental disomy of chromosome 2 Pediatr Neonatol 2020 61 05 558 560 32482602 10.1016/j.pedneo.2020.04.005

[69] Takenouchi T Yamada T Kashiwagi Y Yamaguchi Y Uehara T Kosaki K Hypercoagulopathy associated with uniparental disomy of chromosome 2 J Pediatr Hematol Oncol 2020 42 05 370 371 32487849 10.1097/MPH.0000000000001834

[70] UCLA Clinical Genomics Center Szelinger S Krate J Ramsey K Congenital myasthenic syndrome caused by a frameshift insertion mutation in *GFPT1* Neurol Genet 2020 6 04 e468 32754643 10.1212/NXG.0000000000000468 PMC7357421

[71] Shchagina O Bessonova L Bychkov I Beskorovainaya T Poliakov A A family case of congenital myasthenic syndrome-22 induced by different combinations of molecular causes in siblings Genes (Basel) 2020 11 07 821 32707643 10.3390/genes11070821 PMC7397044

[72] Sezer A Kayhan G Koç A Ergün M A Perçin F E Warburg micro syndrome 1 due to segmental paternal uniparental isodisomy of chromosome 2 detected by whole-exome sequencing and homozygosity mapping Cytogenet Genome Res 2020 160 06 309 315 32599602 10.1159/000509214

[73] Prasov L Ullah E Turriff A E Expanding the genotypic spectrum of Jalili syndrome: Novel CNNM4 variants and uniparental isodisomy in a north American patient cohort Am J Med Genet A 2020 182 03 493 497 32022389 10.1002/ajmg.a.61484 PMC8041260

[74] Kohl S Baumann B Dassie F Paternal uniparental isodisomy of chromosome 2 in a patient with *CNGA3* -associated autosomal recessive achromatopsia Int J Mol Sci 2021 22 15 7842 34360608 10.3390/ijms22157842 PMC8346044

[75] Schüle I Berger U Matysiak U A homozygous deletion of exon 5 of *KYNU* resulting from a maternal chromosome 2 isodisomy (UPD2) causes Catel-Manzke-Syndrome/VCRL syndrome Genes (Basel) 2021 12 06 879 34200361 10.3390/genes12060879 PMC8227568

[76] Knapp A Jagła M Madetko-Talowska A Paternal uniparental disomy of chromosome 2 resulting in a concurrent presentation of Crigler-Najjar syndrome type I and long-chain 3-hydroxyacyl-CoA dehydrogenase deficiency Am J Med Genet A 2022 188 06 1848 1852 35199468 10.1002/ajmg.a.62696

[77] Hara-Isono K Matsubara K Hamada R A patient with Silver-Russell syndrome with multilocus imprinting disturbance, and Schimke immuno-osseous dysplasia unmasked by uniparental isodisomy of chromosome 2 J Hum Genet 2021 66 11 1121 1126 34031513 10.1038/s10038-021-00937-7

[78] Tao Y Han D Wei Y Wang L Song W Li X Case Report: Complete maternal uniparental disomy of chromosome 2 with a novel *UNC80* splicing variant c.5609-4G> A in a Chinese patient with infantile hypotonia with psychomotor retardation and characteristic facies 2 Front Genet 2021 12 747422 34594366 10.3389/fgene.2021.747422 PMC8476880

[79] Li H Wang L Zhang C A rare case of dysferlinopathy with paternal isodisomy for chromosome 2 determined by exome sequencing Mol Genet Genomic Med 2023 11 02 e2110 36464789 10.1002/mgg3.2110 PMC9938747

[80] Lopour M QR Schimmenti L A Boczek N J Kearney H M Drack A V Brodsky M C Alström syndrome caused by maternal uniparental disomy Am J Ophthalmol Case Rep 2022 29 101745 36636630 10.1016/j.ajoc.2022.101745 PMC9829691

[81] Molloy B Jones E R Linhares N D Uniparental disomy screen of Irish rare disorder cohort unmasks homozygous variants of clinical significance in the *TMCO1* and *PRKRA* genes Front Genet 2022 13 945296 36186440 10.3389/fgene.2022.945296 PMC9515794

[82] Nishimura-Kinoshita N Ohata Y Sawai H A case of hyperphosphatemic familial tumoral calcinosis due to maternal uniparental disomy of a *GALNT3* variant Clin Pediatr Endocrinol 2023 32 03 161 167 37362161 10.1297/cpe.2022-0071 PMC10288290

[83] Jain R Rabea F Alfalasi R Elabiary M W Abou Tayoun A Paternal uniparental isodisomy of chromosome 2 in a patient with congenital hypothyroidism: ruling out recessive inheritance or a kinship/laboratory sequencing error J Appl Lab Med 2023 8 05 993 999 37478349 10.1093/jalm/jfad039

[84] Chen Q Chen Y Shi L Uniparental disomy: expanding the clinical and molecular phenotypes of whole chromosomes Front Genet 2023 14 1.232059E6 10.3389/fgene.2023.1232059 PMC10582337 37860673

